# Aging and Senescence of Dental Pulp and Hard Tissues of the Tooth

**DOI:** 10.3389/fcell.2020.605996

**Published:** 2020-11-30

**Authors:** Hidefumi Maeda

**Affiliations:** ^1^Department of Endodontology and Operative Dentistry, Kyushu University, Fukuoka, Japan; ^2^Department of Endodontology, Kyushu University Hospital, Fukuoka, Japan

**Keywords:** aging, dental pulp cells, dental hard tissue, senescence, tooth

## Abstract

The ability to consume a meal using one’s own teeth influences an individual’s quality of life. In today’s global aging society, studying the biological changes in aging teeth is important to address this issue. A tooth includes three hard tissues (enamel, dentin, and cementum) and a soft tissue (dental pulp). With advancing age, these tissues become senescent; each tissue exhibits a unique senescent pattern. This review discusses the structural alterations of hard tissues, as well as the molecular and physiological changes in dental pulp cells and dental pulp stem cells during human aging. The significance of senescence in these cells remains unclear. Thus, there is a need to define the regulatory mechanisms of aging and senescence in these cells to aid in preservation of dental health.

## Introduction

Oral health in older individuals is closely related to their general well-being (Gil-Montoya et al., 2015). In particular, tooth loss causes reduced dietary intake, presumably leading to systemic health problems (Iwasaki et al., 2016). Thus, there is an urgent need for individuals to conserve their teeth in the modern aging society. Tooth fracture is an important cause of tooth loss, which affects 32% of individuals with tooth loss in Japan and 62% of such individuals in Sweden (Axelsson et al., 2004; Yoshino et al., 2015). Other causes include dental caries and periodontal diseases. Because of life extension and increasing focus on oral health care, older individuals possess greater numbers of teeth, as well as a greater risk of tooth fracture, than do younger individuals (Yoshino et al., 2015; Tavares et al., 2018). This may be due to aging-related changes in teeth, including hard and soft tissues. A severely fractured tooth must be extracted. Hence, there is a need to understand alterations of tissues in aged teeth to explore the mechanisms underlying their age-related molecular changes; this will aid in elucidating methods that can prevent tooth loss and support a healthy lifestyle. In this review, I summarize the findings (mainly in the past two decades) concerning aging-related structural alterations of hard tissues, as well as the aging-related molecular and physiological changes in dental pulp cells and dental pulp stem cells (DPSCs). I also discuss methods to counteract tooth senescence for health preservation among older individuals.

## Senescence of Enamel

**FIGURE 1:**
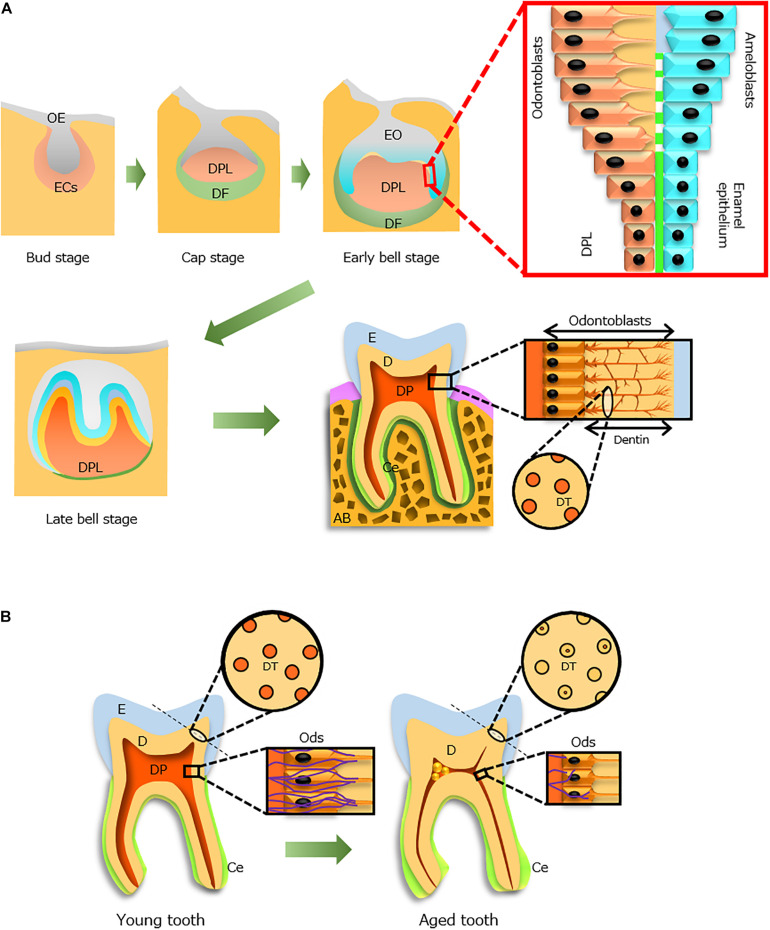
Tooth development during the aging process. **(A)** Tooth development from the bud stage to eruption. Odontoblasts (Ods, orange) and ameloblasts (blue) form dentin (D) and enamel (E), respectively, through epithelial–mesenchymal interactions. An erupted tooth consists of three hard tissues [E, D, and cementum (Ce)] and a soft tissue [dental pulp (DP)]. The processes of Ods extend into the D where dentinal tubules (DTs, circle) are formed. **(B)** Aging alterations of the tooth structure. Constriction of the DP cavity (red), occlusion of DTs in increased D, thickening of the Ce (light green), size reduction of Ods (rectangle), and decreased distribution of nerve fibers (violet, rectangle) with advancing age. AB, alveolar bone; DF, dental follicle; DPL, dental papilla; ECs, ectomesenchymal cells; EO, enamel organ; OE, oral epithelium. Light and dark green indicate Ce and periodontal ligament, respectively.

Enamel that covers the tooth surface is the hardest tissue in the body (Figure 1A) because it comprises >96% minerals (mainly hydroxyapatite crystals). The roles of enamel are to protect internal tissues (e.g., dentin and dental pulp) from caries-causing bacteria, chemo-mechanical attacks, and thermal attacks; it also mediates chewing force.

In the developing tooth, enamel is formed by epithelial cells (ameloblasts) that originate from ectoderm (Figure 1A). These cells interact with mesenchyme-derived dental papilla cells and produce enamel matrix proteins. Initially, the matrix proteins are partially calcified; during enamel maturation, the enamel matrix becomes mineralized, and mature ameloblasts remove both degraded matrix proteins and water to complete the mineralization process (Simmer et al., 2010). At the final stage of differentiation before eruption, ameloblasts exhibit reduced size and no longer contribute to enamel maturation or matrix secretion.

Freshly erupted tooth enamel is more permeable than aged tooth enamel (Bertacci et al., 2007). However, the inorganic components of this tissue can be affected by changes in saliva properties and lifestyle habits during the aging process; in particular, isomorphic and isoionic exchanges lead to increased mineral density (Kunin et al., 2015). During the aging process, enamel is subjected to tooth wear (Bartlett and O’Toole, 2019); it also shows post-eruptive maturation through a reduction in permeability, which leads to greater mineral contents over time, particularly on the outer enamel surface (Park et al., 2008). This results in diminished fracture toughness, partly caused by the reduction of organic matrix (Zheng et al., 2013); it also decreases caries susceptibility (Kotsanos and Darling, 1991). In older individuals, cracks are often observed during clinical examination of the enamel surface, alongside enamel crystals (Yahyazadehfar et al., 2016). However, it is not yet possible to prevent the enhancement of enamel brittleness with age.

## Senescence of Dentin

Dentin is a hard tissue with a unique structure (Figure 1A), which protects and encloses dental pulp tissue in the pulp chamber. It is formed by odontoblasts that differentiate from neural crest-derived ectomesenchymal cells (Figure 1A) and comprises approximately 70% minerals, 18% organic materials, and 12% water. In dentinogenesis, dentinal tubules in dentin are formed by the cell processes of odontoblasts localized at the dental pulp periphery (Figure 1A). Odontoblasts contribute to the sensing of some external stimuli (e.g., caries and tooth cutting) and enhance dentinal resistance against occlusal force.

During the aging process, dentinal sclerosis (i.e., occlusion of dentinal tubules by mineral deposition) progresses (Azaz et al., 1977). The dentin of older individuals shows a higher elastic modulus (Xu et al., 2014) and lower complex modulus, as compared with that of young individuals (Ryou et al., 2015). These structural alterations in older individuals lead to reduced root dentin fracture resistance (Yan et al., 2017).

Odontoblasts are critical cells that form and maintain dentin. They remain alive throughout life but experience aging (Couve, 1986) and undergo deposition of secondary dentin, which increases the hardness of dentin and constriction of the pulp chamber space (Figure 1B; Xu et al., 2014; Carvalho and Lussi, 2017). However, with advancing age, odontoblast densities in human teeth decrease (Daud et al., 2016); moreover, the cell shape of odontoblasts changes from columnar in young individuals to cuboidal in older individuals (Figure 1A; Burke and Samarawickrama, 1995). Aged odontoblasts exhibit greater accumulation of lipofuscin, which is a waste product in lysosomes of older individuals that reduces the thickness of the odontoblastic layer (Figure 1B) and impedes autophagic and dentinogenic activities (Couve and Schmachtenberg, 2011; Couve et al., 2013). Therefore, regulation of odontoblast senescence may allow maintenance of dentin construction in a manner similar to that of young dentin.

## Senescence of Cementum

Cementum is a calcified tissue that comprises approximately 65% minerals, 23% organic materials, and 12% water, which surrounds root dentin and plays a pivotal role in connecting a tooth to its corresponding bone socket via penetration of periodontal fibers (Figure 1A). Thus, cementum is located subgingivally. Cementum is formed by cementoblasts, which differentiate from ectomesenchymal cells; its properties are similar to those of bone (Lehnen et al., 2012). This tissue includes two types: acellular and cellular. The acellular type is present in the coronal and middle portions of the root, while the cellular type is present around the apical portion and encloses cementocytes, such as osteocytes in bone matrix (Ayasaka et al., 1992). However, the features of these cells have not yet been fully elucidated (Zhao et al., 2016).

The characteristic of aging cementum is a continuous increase in thickness, primarily around the root apex (Zander and Hurzeler, 1958; Figure 1B). Additionally, in older individuals, gingival recession significantly increases, as compared with that of young individuals (Hartmann and Muller, 2004); moreover, cementum exposure is evident in older individuals (Barker, 1975). Because the exposed cementum is compromised and has a low resistance to an acidic environment (Shellis, 2010), root caries incidence increases with age (Griffin et al., 2004). Thus, the prevention of gingival recession is essential for tooth maintenance. Thus far, no reports have examined the molecular mechanisms of aging cementum; thus, there is no known mechanism to regulate the thickening of cementum with age.

## Senescence of Dental Pulp

Dental pulp is the soft connective tissue that includes odontoblasts, fibroblasts, mesenchymal stem cells, nerve fibers, and vessels. This tissue is derived from dental papilla, an ectomesenchymal cell condensation in the developing tooth (Figure 1A). The dental papilla cells underlying the enamel epithelium can differentiate into odontoblasts; this process is controlled by epithelial–mesenchymal interactions (Thesleff et al., 2001). Under such epithelial–mesenchymal interactions and the effects of various growth factors (e.g., bone morphogenetic proteins, fibroblast growth factors, and WNT), tooth development proceeds with this tissue (Thesleff, 2003).

The structural relationship between dentin and dental pulp is known as the “dentin–pulp complex.” As mentioned above, the pulp volume decreases with age because of secondary dentin deposition throughout life (Murray et al., 2002). This continuous deposition of dentin and dystrophic calcification in pulpal arteries interrupts blood circulation in the dental pulp of older individuals (Bernick, 1967a). When comparing the cell density of pulp in 70-year-old individuals with that in 20-year-old individuals, the cell number in older individuals was nearly half that of the younger individuals (Nanci, 2018), indicating a reduction in pulp restoration activity (Murray et al., 2002). Additionally, Hillmann and Geurtsen (1997) reported that the collagen fiber bundle aggregation and calcification in dental pulp increased with advancing age. Dystrophic calcification in the central pulp of the coronal region and root canal is evident in older individuals, which might be due to their reduced pulpal blood flow (Ersahan and Sabuncuoglu, 2018; Iezzi et al., 2019). Notably, Li et al. (2011) reported that human dental pulp cells (HDPCs) cultured under hypoxic conditions exhibited increased mineralization. Furthermore, a previous study examined the morphological alteration of the dentinal pulp wall during the aging process (Tsurumachi et al., 2008); the shape and modality of calcospherites on the pulp wall became diverse during aging. Such deposition may contribute to the growth of secondary dentin.

Tertiary dentin also deposits on secondary dentin under pathological stimuli such as dentin caries, tooth cutting, and trauma. This dentin includes two types, reactionary and reparative, which differ depending on the degree of stimuli (Smith et al., 1995). These dentinogenic activities are also reduced in older individuals (Murray et al., 2002).

With age, difficulties in endodontic treatment occur due to the constriction of pulp chamber space by hyperplasia of secondary and tertiary dentin, as well as pulp stones (i.e., ectopic calcified particles in the coronal region) and diffuse calcification in radicular pulp (Krasner and Rankow, 2004). A recent study reported the efficiency of cone-beam computed tomography in endodontic diagnosis and treatment planning (Sue et al., 2018), demonstrating its usefulness in current treatment. Additionally, guided endodontic access using cone-beam computed tomography in patients with calcified root canals has been described (Lara-Mendes et al., 2018).

Nerve fibers are widely distributed in dental pulp. In the growing dental papilla of human fetal teeth, expression of nerve growth factor and its low and high affinity receptors (p75NTR and TrkA, respectively) precede the initiation of tooth innervation (Mitsiadis and Pagella, 2016). In particular, p75NTR is thought to condense mesenchymal cells from neural crest cells during the construction of dental papilla. Therefore, these molecules are presumably involved in both tooth development and nerve growth in dental pulp. However, the distribution of nerve fibers in dental pulp decreases, probably because of degeneration with increasing age (Bernick, 1967b; Figure 1B). Notably, Couve and Schmachtenberg (2018) characterized two types of Schwann cells that reside in dental pulp: non-myelinating and myelinating. They also found a reduction in the network of these cells at the dentin–pulp interface, along with decreased innervation in old dental pulp; these findings suggested the progression of less symptomatic caries in older individuals because of a reduced response to environmental injuries and pathogens (Couve et al., 2018). Thus, during aging, the pulp cavity constricts and dental pulp cells subsequently reduce their functions and activities (Figure 1B).

## Senescence of Dental Pulp Cells and Dental Pulp Stem Cells

Cellular senescence, characterized by irreversible arrest of cell proliferation, is evoked by various intrinsic and extrinsic stressors such as DNA damage, oxidative stress, telomere damage, oncogene activation and/or inactivation, and spindle stress (Herbig et al., 2006; van Deursen, 2014). Currently, senescence is viewed as a multistep process comprising biological activity and evolution. The roles of senescent cells in aging have been discussed widely. Briefly, there are two categories of senescence: acute and chronic (van Deursen, 2014). Acute senescence is induced by acute and specific stress, targets specific cells, and is a scheduled process; this type of senescence is involved in normal biological processes (e.g., tissue repair, development, and wound healing). Chronic senescence is promoted by gradual increases in stress and damage, which have detrimental effects on nearby cells; moreover, it does not target specific cells and is an unscheduled process (Dodig et al., 2019). This pathway is considerably different from acute senescence due to the heterogeneity of senescence-associated secretory phenotype (SASP) factors that include cytokines, chemokines, proteases, growth factors, and matrix metalloproteases (Byun et al., 2015).

During the senescence process, senescent cells secrete SASP factors (Coppe et al., 2008) that affect adjacent cells in autocrine or paracrine manners (Acosta et al., 2013; Borodkina et al., 2018). SASP factors play pivotal roles in the above two senescent modalities; these factors have beneficial or detrimental effects on cellular senescence, such as regeneration, degeneration, immune clearance, or growth stimulation (Watanabe et al., 2017). Acute senescent cells are removed by immune cells following stimulation by activated SASP factors; various SASP factors induce chronic inflammation (Dodig et al., 2019), which results in age-associated disorders and tumorigenesis. Although the main roles of SASP factors are to preclude senescent cells by immune clearance, the effectiveness of this process is influenced by age-associated changes that hinder the immune system (Denkinger et al., 2015).

In this context, to prevent cellular senescence, two possible therapies of anti-senescence have been proposed: suppression of SASP factors or selective elimination of senescent cells (Watanabe et al., 2017; de Magalhaes and Passos, 2018). Although these are regarded as promising strategies, there is a need to carefully consider whether targeting SASP factors is a suitable method because these factors have both favorable and unfavorable characteristics.

### Senescence of Dental Pulp Stem Cells

Dental pulp stem cells (Gronthos et al., 2000) have attracted considerable attention as promising cells for regenerative endodontics (Murray et al., 2007) and systemic regenerative medicine (Anitua et al., 2018; Yoshida et al., 2020). This attention has arisen because DPSCs exhibit characteristics of mesenchymal stem cells that can differentiate into various cell types such as odontoblastic cells, dental pulp cells, neuronal cells, vascular endothelial cells, retinal cells, islet cells, hepatocytes, smooth muscle cells, osteoblastic cells, adipocytes, and chondrocytes (Anitua et al., 2018; Yoshida et al., 2020). Additionally, during acquisition of DPSCs from patients, invasive stress is very low relative to stem cell isolation from other organs and tissues. Thus, cell banking has been implemented for on-demand transplantation because human DPSCs (HDPSCs) reside in dental pulp of young individuals at proportions of 0.67–1.02% (Honda et al., 2007). A recent study found the expression of CD24a in multipotent stem cells derived from human dental papilla in a developing tooth germ, which developed into dental pulp and then differentiated into odontoblasts in the mature tooth (Chen et al., 2020). The number of CD24a(+) cells in dental pulp remains unclear, but this discovery might enable efficient isolation and expansion of a pure stem cell population.

These stem cells also undergo aging. The proliferation and differentiation capacities of HDPSCs are impaired in older individuals (Yi et al., 2017). However, HDPSCs show reduced cellular senescence, as well as enhanced osteogenesis and proliferation activities, compared with bone marrow mesenchymal stem cells, periodontal ligament stem cells, and adipose-derived stem cells (Ma et al., 2019).

**FIGURE 2:**
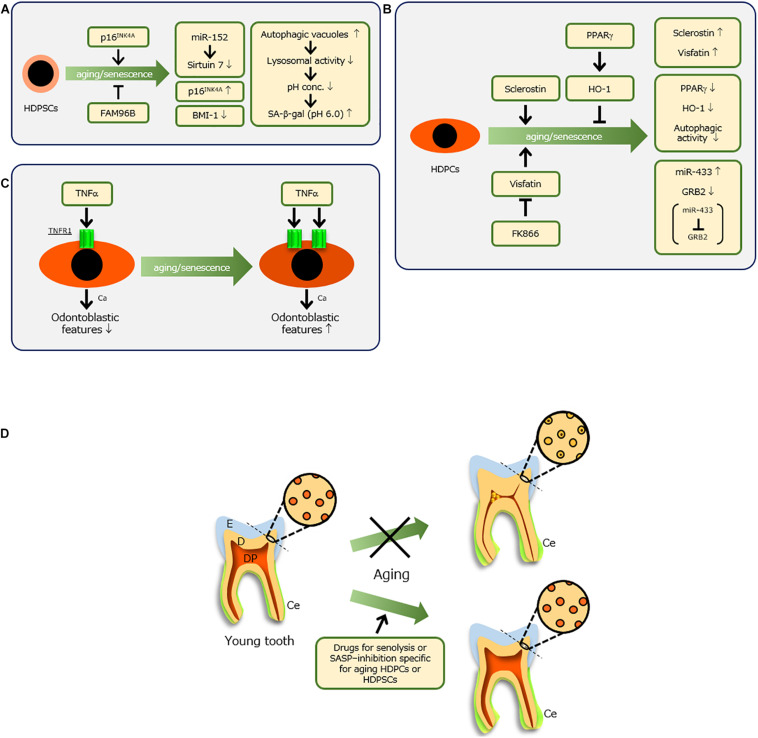
Molecules involved in senescence of human dental pulp stem cells (HDPSCs) **(A)** and human dental pulp cells (HDPCs) **(B)** and their molecular features. **(C)** When senescent HDPCs with increased expression of TNF receptor 1 (TNFR1) were treated with tumor necrosis factor-alpha (TNF-α) and calcium (Ca), their odontoblastic features were enhanced, whereas young HDPCs showed reductions of these features. **(D)** Ideal therapy to preserve a tooth with advancing age. When dental pulp (DP) and dentin (D) are maintained [e.g., by drug-related induction of a non-constrictive DP space and non-occluded dentinal tubules for senolysis or senescence-associated secretory phenotype (SASP) inhibition, specifically targeting aging HDPCs or HDPSCs], healthy teeth might be maintained throughout life. Because no report has examined the molecular mechanism of aging cementum (Ce), it is difficult to prevent thickening of Ce, which does not affect tooth fracture. E, enamel.

Various studies have examined the mechanisms of changes in HDPSCs during aging (Figure 2A). One study reported that senescent HDPSCs have decreased BMI-1 expression but increased p16^*INK*4*A*^ expression; overexpression of BMI-1 in senescent HDPSCs rescued senescence-impaired odontogenic differentiation (Mehrazarin et al., 2011). Feng et al. (2014) reported that the activation of p16^*INK*4*A*^ signaling stimulated onset of senescence in HDPSCs. Another report demonstrated that p16^*INK*4*A*^ and BMI-1 are involved in the senescence of HDPSCs induced by oxidative stress (Mas-Bargues et al., 2017). However, Gu et al. (2016) showed that Sirtuin 7 is downregulated in senescent HDPSCs through identification of Sirtuin 7 as a target of miR-152, an inducer of senescence in HDPSCs. Another recent notable study showed that HDPSCs from older individuals have decreased expression of the family with sequence similarity 96 member B homeobox (FAM96B) gene; overexpression of FAM96B improved their proliferation and differentiation abilities, whereas it downregulated senescence markers such as senescence-associated-β-galactosidase (SA-β-gal), p16, and p53 (Liang et al., 2020).

Human DPSCs undergoing replicative senescence exhibit increased numbers of autophagic vacuoles (Li et al., 2012; Figure 2A). This suggests reduced lysosomal activity due to the accumulation of autophagic vacuoles (Rezzani et al., 2012), which indicates lipofuscin accumulation in lysosomes (Couve et al., 2013). When lysosomal activity decreases in senescent cells, the proton concentration decreases (Colacurcio and Nixon, 2016). Therefore, the optimal pH of lysosomal β-galactosidase is 4, whereas that of SA-β-gal in lysosomes (a biomarker of cell senescence) is 6 (Dimri et al., 1995). Hence, a pH probe is under investigation as a candidate anti-senescence therapy for mesenchymal stem cells (Wang et al., 2018). In this context, Morsczeck (2019) has attempted to clarify the mechanism involved in regulating DPSC senescence, which is important for optimizing related therapeutic applications in regenerative medicine.

Although the above findings may provide methods to prevent tooth aging and promote pulp regeneration, further analyses are needed to integrate these data.

### Senescence of Dental Pulp Cells

All cells in dental pulp are generically regarded as dental pulp cells, but they mainly comprise fibroblastic cells. Biological and molecular alterations of HDPCs with age have been reported (Figure 2B). With advancing age, HDPCs show reductions in proliferation and alkaline phosphatase activity, thereby indicating impaired restoration of injured pulp tissue (Shiba et al., 2003). Sclerostin produced by osteocytes suppresses osteogenesis (Moester et al., 2010), but its inhibition induces bone formation (Zhang et al., 2020). Sclerostin also inhibits odontoblastic features of HDPCs (Liao et al., 2019), whereas deficiency of sclerostin facilitates reparative dentin formation *in vivo* (Figure 2B; Collignon et al., 2017). Notably, Ou et al. (2018) reported that HDPCs from older individuals have increased sclerostin production, while overexpression of sclerostin in young HDPCs induces senescent features (Figure 2B).

Additionally, it was recently elucidated that visfatin, a type of adipokine, increases in senescent HDPCs; exogenous visfatin induces HDPC aging along with upregulation of SASP factors, whereas the chemical inhibitor of visfatin, FK866, reduces senescent features in HDPCs (Figure 2B; Ok et al., 2020). Another group reported that miR-433, which is increased in aged HDPCs, is a senescence-associated miRNA of HDPCs that targets GRB2 (Figure 2B; Wang et al., 2015). Importantly, GRB2 undergoes tyrosine phosphorylation during T-cell aging (Ghosh and Miller, 1995; Chang et al., 2019).

Yi et al. (2017) examined the involvement of peroxisome proliferator-activated receptor gamma (PPARγ) and its downstream effector heme oxygenase 1 in aging HDPCs (Figure 2B; Lee et al., 2013b). PPARγ functions in the survival and differentiation of HDPCs and protects against oxidative stress through heme oxygenase 1 (Lee et al., 2013a). Lee et al. (2015) reported that senescent HDPCs showed reductions in these molecules, while increased SA-β-gal activity and decreased PPARγ influenced autophagic activity and cellular homeostasis in senescent HDPCs. The regulation of these two molecules might be important in tooth conservation in older individuals.

In this context, we focused on tumor necrosis factor-alpha (TNF-α), a known SASP factor (Figure 2C). In our recent study, we compared odontoblastic differentiation of HDPCs at low replicative senescence [population doubling (PD) 6] with HDPCs at high replicative senescence (PD28); HDPCs at PD28 expressed senescence makers such as p16, p21, p53, and SA-β-gal (Nozu et al., 2018). Similar to other reports, senescent HDPCs showed reduced differentiation activity. However, treatment of senescent HDPCs with TNF-α resulted in higher odontoblastic differentiation activities and mineralization per cell, compared with HDPCs at PD6. These results were related to higher expression levels of TNF receptor 1 (TNFR1) in senescent HDPCs, compared with those levels in younger HDPCs (Nozu et al., 2018). Importantly, senescent HDPCs treated with TNF-α and calcium demonstrated increased odontoblastic features, whereas young HDPCs showed reduction of such features (Figure 2C; Nozu et al., 2018). This was consistent with a recent study in which HDPCs exhibited enhancement of mineralization potential with increasing passage (Bakopoulou et al., 2017). In older individuals, ectopic calcification is often observed in dental pulp, characterized by pulp stones that narrow the pulp chamber space, as well as diffuse calcification in radicular pulp (Bernick, 1967a; Nitzan et al., 1986; Iezzi et al., 2019). As teeth are exposed to long-term stress (e.g., mastication, occlusion, or bruxism), these weak and chronic inflammatory conditions might lead to induction of SASP factors in HDPCs, thereby causing age-related alterations. Overall, because HDPCs and HDPSCs regulate dental pulp senescence, local control of senescence progression might conserve teeth.

## Conclusion

This review provided an overview of the findings over the past two decades regarding the senescence of hard and soft tissues in teeth. However, this issue has a short history in dentistry. In the modern aging society, it is important to explore methods to counteract the senescence of teeth, particularly HDPCs and HDPSCs, to aid in tooth conservation. As mentioned above, the development and application of drugs for senolysis or SASP inhibition, specifically targeting aging HDPCs or HDPSCs, are an extremely attractive approach. For success with this method, the method by which senescent cells are targeted is a crucial factor. This issue must be resolved to overcome the health problems affecting the modern aging society. Consequently, maintenance of dental pulp and dentin in a manner similar to that of young teeth will protect against tooth loss by averting tooth fracture, thereby preserving dental health (Figure 2D).

## Author Contributions

The author confirms being the sole contributor of this work and has approved it for publication.

## Conflict of Interest

The author declares that the research was conducted in the absence of any commercial or financial relationships that could be construed as a potential conflict of interest.
